# Supplementary material for: Contrasting effects of SARS-CoV-2 vaccination vs. infection on antibody and TCR repertoires
Source: PLoS One. 2026 Mar 27;21(3):e0343939. doi: 10.1371/journal.pone.0343939 (PMC13028327; doi:10.1371/journal.pone.0343939)
Supplement: Attachment — Submitted filename: 149380_1_rebuttal_3405978_sxfxdg.pdf [file pone.0343939.s002.pdf]

We thank the reviewers for their careful and thoughtful read of our work.

Reviewer 1 helpfully suggested that the audience might not know what fuzzy matching is. The manuscript contains the following explanation of fuzzy matching:

"That subjects had almost no exact matches to SARS-CoV-2-specific IGH sequences did not exclude the possibility that they have sequences that are functionally similar to these reference sequences. The same possibility exists for TRBs. A standard method for finding similar sequences is using the Levenshtein (edit) distance. Sequences with a distance of less than or equal to a tolerance t are considered similar (for example, sequences that differ by no more than t=1 amino acid). This is known as "fuzzy matching" with tolerance t. (Note that exact matches are just fuzzy matches with tolerance 0.)"

We now also add the word "approximate" in conjunction with earlier uses of the word "fuzzy."

Reviewer 2 asked whether we "focused on potential contributions [to CDR3 length variations] based on germline gene usage, rather than directly observed contributions from the V and J segments within the CDR3 regions;" the answer is, the latter. Reviewer 2 also pointed out that it would be valuable to have HLA typing for a more comprehensive analysis. We wholeheartedly agree and have added a sentence to this effect in the discussion.

Reviewer 3 had several specific comments. The first was regarding the overall implication of the study. There are several:

- binding capacity is as predictive as, and more robust than, prior approaches. As we write: "We found that repertoires' binding capacity to known SARS-CoV-2-specific CD4+ TRBs performs as well as the best hand-tuned approximate or "fuzzy" matching at predicting a protective level of NAbs, while also being more robust to repertoire sample size and not requiring hand-tuning."

- the importance of looking for unexpected patterns, for example in non-productive joins as was done here, and for global small-scale perturbations that together result in unavoidable signals. As we write, "B- and T-cell adaptive responses to SARS-CoV-2 infection and vaccination are surprising, subtle, and diffuse," and "One open question is to what extent infection affects antibody and TCR repertoires as a whole vs. enriching specific clones within it. One can refer to these ends of the continuum of possible effects as "diffuse" vs. "precise.""

- caution against over-interpreting correlations with specific gene segments and. As we write: "With these caveats in mind, to our knowledge previous studies have identified 20

IGH V genes to be enriched in sequences produced during various immune responses to SARS-CoV-2.8–16  Given that human genomes encode 54 IGH V genes,17 collectively these studies implicate 37% of V genes in the response to this single viral exposure, indicating that the SARS-CoV-2 response is either quite broad within individuals, quite heterogeneous among individuals, or both."

Each of these challenges prevailing approaches, understanding, and conclusions about patterns and signatures in repertoire sequence. We should hope this would be of some benefit.

Reviewer 3 also asked what type of vaccines the participants received. We have now clarified that they all received an mRNA vaccine: 80% receiving Pfizer Comirnaty and the rest receiving Moderna Spikevax.

We looked at anti-spike neutralizing antibodies because this is where the evidence for neutralization is strongest. It would have been great to have diagnostics for every protein as well as Fc function, but these were not available and therefore not possible to study.

Reviewer 3 noted that we mention that it is impossible to know a priori what study size would be adequate to identify public sequences comprehensively in COVID-19 and asks if 251 individuals are enough. Assuming this is in reference to the size of our study, we would like to point out that this study does not claim to identify public sequences comprehensively. The rationale is more is better. The statistics tell the reader the extent to which to reject the null hypotheses put forth.

Regarding comorbidities: we probably could perform an analysis on their impact in a future study. We thank the reviewer for this idea.

Regarding timing: samples were collected from the vaccinee cohort 4 to 84 days (mean = 44.3 days, standard deviation = 15.3 days) after administration of the initial vaccine dose. Supplementary Figure S13 shows sampling times vs. NAb titers.

We feel the length of the introduction is required to contextualize the implications and benefits of this study.

We were unable to find the typos referred to but did run the manuscript through spelling and grammar checks again. We thank the Reviewer for the thoughtful attentiveness.
