# Supplementary material for: Contrasting effects of SARS-CoV-2 vaccination vs. infection on antibody and TCR repertoires
Source: PLoS One. 2026 Mar 27;21(3):e0343939. doi: 10.1371/journal.pone.0343939 (PMC13028327; doi:10.1371/journal.pone.0343939)
Supplement: Attachment — Submitted filename: Response to Reviewers.pdf [file pone.0343939.s003.pdf]

Reviewer comments in *italics* in **black;**, replies in non-italics in **blue**.

Reviewer #1:

Overall the data is valuable and the background is well-explained. However, the machine learning section for predicting NAb titer is relatively simplistic and lacks sufficient clarity. In particular, it is not clear how features based on exact and fuzzy sequence matching are generated from the repertoire data. It would be helpful to include a clear framework diagram illustrating the overall modeling pipeline, along with a small example of input data to show what the features actually look like. This would greatly improve understanding of how repertoire data is transformed into model inputs.

We have now added a new supplemental figure S11 with such a diagram of how repertoire data is transformed into model inputs, including an illustration of how fuzzy matching works.

The use of NAbs as a functional readout makes sense for BCR, but it does not directly reflect whether TCR repertoires undergo antigen-specific changes. Some individuals may have strong T-cell responses but weak antibody responses, especially mild or asymptomatic cases or in some vaccinated individuals.

The connection is indirect but nonetheless solid. T-cell help is essential to generating NAbs. Antigen-specific T helper cells in germinal centers drive the class switching, somatic hypermutation, and B-cell proliferation that lead to NAbs production. Conversely, NAbs are almost never observed in the absence of specific T-cell help. That specificity depends on TCR. So while what the reviewer says is true, the connection between TCR repertoire and NAbs is strong, and is likely the biological cause of what we observe.

Figure 1 raises questions—NAb titers appear higher in vaccinees than infectees, particularly in younger individuals, and the difference is significant. Is this biologically reasonable? More explanation is needed.

Yes: vaccines are engineered to boost NAb titer, unlike natural infection. Vaccines are are designed to present the most neutralization-sensitive epitopes, are delivered together with adjuvants, and administered in multiple doses—all things explicitly calculated to boost NAbs.

Some references only include DOIs without journal names. Please ensure references are complete.

These have been corrected.

Reviewer #2:

In the abstract line 31-33: the authors state that: "Vaccination and infection have an effect on non-productively recombined IGHs, suggesting an effect that precedes clonal selection." This statement is confusing. It is better to say what the effect is rather than stating that there is an effect.

We have modified these lines to now say “vaccination and infection are associated with longer non-productively recombined IGHs.”

In the abstract line 33-36: the statement “We found that repertoires’ binding capacity to known SARS-CoV-2 specific CD4+ TCRB performs as well as the best hand tuned approximate or fuzzy matching at predicting a productive level of Nabs, while also being more robust to repertoire sample size and not requiring hand tuning.” The statement is not clear. I recommend they rewrite it and explain what repertoires’ binding capacity they are referring to? T cell or B cells?

We have clarified that this statement refers to TRB repertoires.

In the abstract line 38: What do they mean by surprising, subtle, and diffuse?

We believe this is appropriate for the limited space of an abstract. The “longer non-productively recombined IGHs” previously mentioned will strike knowledgeable readers as surprising. The statements are explained in the body of the manuscript, particularly the Discussion.

Major comments:

1. What is the biological or immunological relevance of the findings? This is not clear in the manuscript. It is unclear what the biological significance of all their bioinformatic analysis is. Can they predict what IGH and TCRB are responsible for the most neutralizing Abs?

The primary biological significance is demonstrating that infection and vaccination can have effects beyond increasing the frequencies of specific IGH and TRB sequences, for example the effects on non-productive sequences noted in the abstract and the changes in binding capacity, which is an emergent, repertoire-level property—”diffuse” was the word we used in the abstract—that need not result from a specific IGH or TRB sequence.

2. They are insinuating that BCR/TCR rearrangement is affected by Ags, while the 2 processes are known to be independent. They do not provide genetic or biologic proof for that, other than there is an increase in non-productive receptor recombination by sequencing. Does infection decrease NHEJ enzymes and TdT, which are important for receptor modification at the junctions and their fusion?

The data say that infection and vaccination affect this upstream process. This was surprising to us as well. We do not know the mechanism, and our laboratory is not configured for mechanistic studies. Science is about communication. It does not wait for the “final word.” We decided not to withhold this finding. We discuss potential mechanisms in the Discussion.

3. The results has a lot of educational information that is too complex to understand by immunologists. It should be focused on the results and their significance.

It is difficult to comment without knowing more precisely what information is being referred to. It is the authors’ opinion that the information presented is necessary for the reader to understand the motivation behind the results and their significance.

4. The discussion is too long and repeats and expands concepts that are introduced in the introduction. It should be focused on discussing their results and compare/contrast them to other studies in the literature.

Several previous reviewers have made the opposite comment, requesting the Discussion be lengthened, resulting in the Discussion in the present manuscript. We think this is a matter of personal preference that will not interfere with comprehension of the work for readers who might prefer less, and enhancing its comprehension for readers who prefer more.

5. The authors obtained their DNA from B and T cells isolated from peripheral blood. B cells with longer IGH CDR3s in non-productive joins are negatively selected because they do not express a productive BCR, and, thus, do not egress out of the bone marrow into the periphery. How do the authors explain obtaining them from peripheral blood? Could they be sequencing the B cells that have one non-productive allele, and thus reinitiated VDJ recombination resulting in a productive recombination of the second allele? In that case, their data is a mix of sequences from productive and non-productive alleles sometimes originating from the same B cell clones.

The non-productive joins are "bystanders" carried along in cells that also contain a productive join. Viable cells contain either one productive join, or else one productive and one non-productive join. Cells that contain two non-productive joins are non-viable and therefore not observed. Because recombination is random (to a very good approximation), there is no meaningful correlation between the nonproductive join and the subsequent productive join from the same cell. The only correlation is the amplification of the non-productive join in cells where the productive join produces a protein that causes the cell to be selected and clonally expand. The advantage of studies as large as ours is, such bystander effects get averaged out by the large number of cells per subject, and the large number of subjects in the study. This is not the explanation for our findings.

6. They only focus on type of CDRs and length of mismatch at the joins, but they do not account for the type of nucleotide at the joins that results in a different amino acid and thus a different Ab variable domain.

This is true, but would be a substantial amount of additional investigation. For this reason, it is left for future work.

7. Line 213: do they mean "Thus selection for "longer" CDR3s in…"

No, "shorter" is correct. To clarify, we have now added "in productive joins."

8. Line 230: correct the English.

The word "be" was added to correct the English.

9. Line 282-283: The statement: "Thus, fuzzy matching was shown to not be a robust measure of repertoire content in this study." is written in poor English. In addition, the authors write this complex paragraph to explain the principle of fuzzy matching to readers who are not expert in the field. Yet at the end they find negative correlation with fuzzy matching as a measure of

robust repertoire analysis. Why not omit this information and just mention using the binding capacity method?

The reason is that fuzzy matching is a reference method, whereas binding capacity is new. Unfortunately, just because it is a reference method doesn't mean all readers will be familiar with it. In addition, even readers familiar with fuzzy matching are unlikely to be aware of its limitations, which our presentation makes clear. Therefore it was necessary to include this information, despite the superiority of binding capacity. We have edited the English as follows: "Thus, fuzzy matching is not a robust way to measure repertoires, at least in this study."
